# Original Fluorinated Non-Isocyanate Polyhydroxyurethanes

**DOI:** 10.3390/molecules28041795

**Published:** 2023-02-14

**Authors:** Lolwa Haydar, Wassim El Malti, Vincent Ladmiral, Ali Alaaeddine, Bruno Ameduri

**Affiliations:** 1 ICGM, University of Montpellier, CNRS, ENSCM, 34095 Montpellier, France; 2 College of Engineering and Technology, American University of the Middle East, Egaila 54200, Kuwait

**Keywords:** aminolysis, cyclocarbonates, fluorinated polymers, polyhydroxyurethanes, SDG3

## Abstract

**Highlights:**

**What are the main findings?**

- Fluorinated telechelic bis(cyclocarbonate) was synthesized through carbonylation of a fluorinated diepoxide.
- Fluorinated polyhydroxyurethanes (FPHUs) with different molar masses were synthesized through polyaddition reaction.
- Analysis showed that the FPHUs have higher molar mass, glass transition, and decomposition temperatures compared to their hydrogenated homologues.

**What is the implication of the main finding?**

- New non-isocyanate polymers with improved properties.
- The synthesized FPHUs are potential candidates for applications requiring materials with these properties (E.g., adhesives).

**Abstract:**

New fluorinated polyhydroxyurethanes (FPHUs) with various molar weights were synthesized via the polyaddition reaction of a fluorinated telechelic bis(cyclocarbonate) (bis-CC) with a diamine. The fluorinated bis-CC was initially synthesized by carbonylation of a fluorinated diepoxide, 1,4-bis(2′,3′-epoxypropyl)perfluorobutane, in the presence of LiBr catalyst, in high yield. Then, several reaction conditions were optimized through the model reactions of the fluorinated bis-CC with hexylamine. Subsequently, fluorinated polymers bearing hydroxyurethane moieties (FPHUs) were prepared by reacting the bis-CC with different hexamethylenediamine amounts in bulk at 80 °C and the presence of a catalyst. The chemoselective polymerization reaction yielded three isomers bearing primary and secondary hydroxyl groups in 61–82% yield. The synthesized fluorinated CCs and the corresponding FPHUs were characterized by ^1^H, ^19^F, and ^13^C NMR spectroscopy. They were compared to their hydrogenated homologues synthesized in similar conditions. The gel permeation chromatography (GPC), differential scanning calorimetry (DSC), and thermogravimetric analysis (TGA) data of the FPHUs revealed a higher molar mass and a slight increase in glass transition and decomposition temperatures compared to those of the PHUs.

## 1. Introduction

Polyurethanes (PUs), which have been known for nearly 70 years, are used in various fields, such as formulations of adhesives, paints, foams, coatings, packaging components, fibers, and in some specialized applications, including biomedical surgeries where biocompatible, biodegradable, and non-toxic PUs are used. PUs have several interesting properties, such as excellent adhesion, high durability, good flexibility, resistance to corrosion and mechanical wear, and favorable optical properties [1].

The conventional method for the production of PUs is based on the reaction between isocyanates and the hydroxyl groups of oligomers [2]. It is a reaction consisting of the polyaddition of a polyisocyanate with polyols in the presence of a labile hydrogen atom. The rigidity of the final product changes depending on the nature of polyols and isocyanates.

The major problem of this chemistry is related to the toxicity and volatility of isocyanates [3,4]. The isocyanates are represented by two main families of aromatic structures: toluene diisocyanate (TDI), and methylene diphenyl diisocyanate (MDI) [5]. The NCO functional groups are introduced into the aromatic nuclei by phosgenation of toluene diamine to prepare TDI or aniline to obtain MDI. Nevertheless, in its turn, phosgene is a toxic reactive precursor involved in a dangerous reaction: phosgenation [6]. Therefore, the “isocyanate route” threatens the environment and operators’ health. Replacing or minimizing these hazardous substances has become both academic and industrial concerns [7].

**Figure 1:**
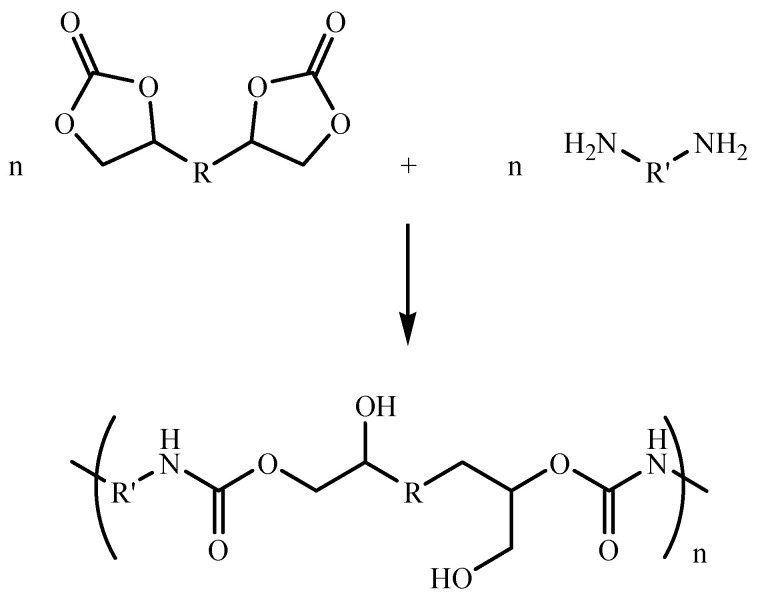
Synthesis of PHUs by polyaddition of CCs and diamines.

Various approaches to the synthesis of isocyanate-free PUs were explored [8]. Among the most common and studied routes, ring-opening polyaddition of cyclocarbonates (CCs) and amines in bulk or solution is an excellent alternative to the conventional PUs based on isocyanate precursors [6,9,10,11,12,13]. The nucleophilic addition of the amines to the CCs leads to polyhydroxyurethanes (PHUs), also called non-isocyanate polyurethanes, which possess urethane linkages and primary and secondary hydroxyl groups that are reported to enhance the adhesive properties of the polymer (Figure 1) [14,15,16].

In general, PHUs showed better chemical, thermal, and mechanical stability than PUs. In comparison to PUs, PHUs have a hydroxyl group adjacent to each carbamate group, which greatly enhances the concentration of hydrogen bonds through intra- or intermolecular interactions. The latter can strongly improve the mechanical and thermal properties and, therefore, the adhesive properties of these polymers [17]. Furthermore, the synthesis process of PHUs overcomes a critical drawback in synthesizing PUs: insensitivity to humidity [18]. On the other hand, the amines required for this aminolysis reaction are commercially available or can be yielded from bio-based resources. The CCs are considered superior to other reagents due to their ease of synthesis, high boiling point, biodegradability, and low toxicity [18,19,20,21]. To the best of our knowledge, only five-membered ring CCs are commercially available. In addition, five- and six-membered ring CCs were successfully and efficiently synthesized and reported in the literature [3,22,23]. The most common routes for synthesizing CCs with excellent selectivity and yields generally involve the insertion of carbon dioxide in epoxides [24,25,26,27,28,29]. Nevertheless, the significant downside in synthesizing PHUs is often the difficulty in reaching high molar masses [30]. Moreover, the polyaddition reaction is slow at room temperature and requires careful control of the stoichiometry of the reactants [31].

In terms of selectivity, the nucleophilic addition of the amine to five-membered CCs leads to the formation of two isomers [32]. However, a single product is obtained using six-membered CCs. In the case of five-membered CCs, both resulting isomers can be distinguished from one another by the presence of a primary or a secondary hydroxyl group [33,34]. Generally, the reaction of CCs with amines favors the formation of the isomer containing the secondary hydroxyl group [35]. As for the case of the 6-membered ring, only one hydroxyurethane product containing primary hydroxyl groups can be obtained [10].

Ultimately, fluorinated polymers (FPs) are reported to demonstrate remarkable properties, including their chemical, thermal, and water resistance [36,37,38,39,40]. These properties are indispensable for multiple industries [41,42], especially for coatings. Most FPs are synthesized by radical (co)polymerization of fluoroalkenes. Two branches of FPs can be considered: (i) FPs, where the fluorinated groups are located within the polymer backbone, and (ii) FPs containing a fluorinated dangling group [39]. To the best of our knowledge, despite the synthesis and characterization of fluorinated PUs, fluorinated polyhydroxyurethanes (FPHUs) were rarely reported in literature. In 2014, FPs bearing five-membered CC pendant groups were synthesized by co- or ter-polymerization of chlorotrifluoroethylene with various vinyl ethers [36]. In 2019, fluorine-containing non-isocyanate polyurethane coatings were synthesized using bisphenol and perfluorooctyl CCs [37].

**Figure 2:**
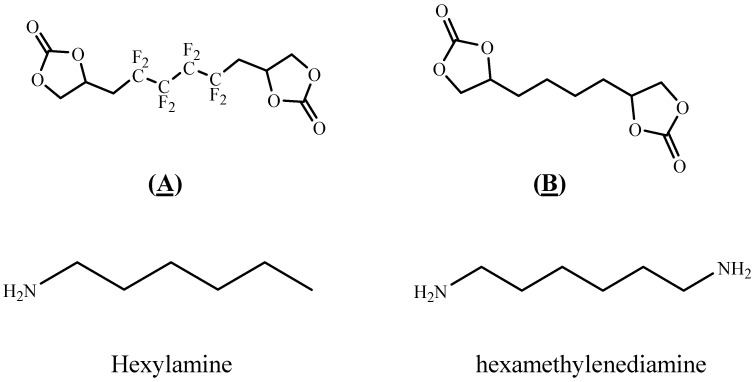
The fluorinated and non-fluorinated biscyclocarbonates (**A** & **B**) and amines used in this work.

In this study, FPHUs were prepared via the polyaddition route, characterized by spectroscopy, and thermally analyzed. For this purpose, fluorinated telechelic bis-CC (Figure 2) derived from 1,4-bis(2′,3′ epoxypropyl)perfluorobutane was synthesized and reacted with mono- and diamine (Figure 2). The optimal reaction conditions were examined by synthesizing model fluorinated hydroxyurethanes from fluorinated bis-CC and hexylamine. Then, FPHUs were formed by reacting the fluorinated bis-CC with hexamethylenediamine using different molar ratios to provide a range of polymers of various molar masses. In light of this work, we synthesized PHUs using the same polymerization conditions and through a synthesized hydrogenated bis-CC (Figure 2). Finally, we compared the properties of both prepared polymers, FPHUs and PHUs.

## 2. Results and Discussion

The syntheses of the fluorinated and non-fluorinated bis-CCs and the subsequently optimized syntheses of FPHUs and PHUs by addition with amines were achieved. The resulting products were characterized by spectroscopy and thermal analyses. In this work, the PHUs were prepared for properties comparison purposes.

### 2.1. Synthesis of Fluorinated Biscyclocarbonate (***A***) and Hydrogenated Biscyclocarbonate (***B***)

**Figure 3:**
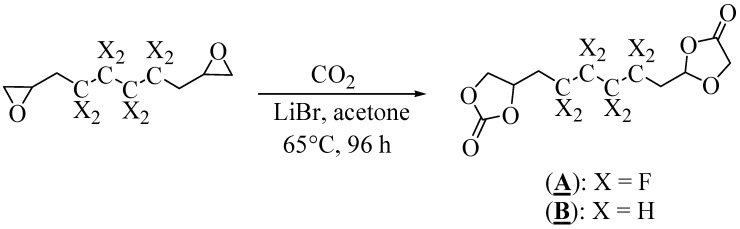
Synthesis of (**A**) and (**B**) by carbonylation of the corresponding diepoxide.

The syntheses of (**A**) and (**B**) were performed by direct carbonylation of the corresponding diepoxide with CO_2_ in the presence of a lithium bromide (LiBr) catalyst (Figure 3) [43].

After optimization of the reaction conditions, the carbonylation was conducted in a reactor in the presence of acetone at 65 °C for 96 h (Table 1). The fluorinated and non-fluorinated precursors (**A**) and (**B**) were then characterized and confirmed by ^1^H, ^13^C (and ^19^F for **A**) NMR spectroscopy (Figures S1–S5 in the Supplementary Materials).

### 2.2. Model Reaction of (***A***) with Monoamine: Optimization of the Reaction Conditions

**Figure 4:**
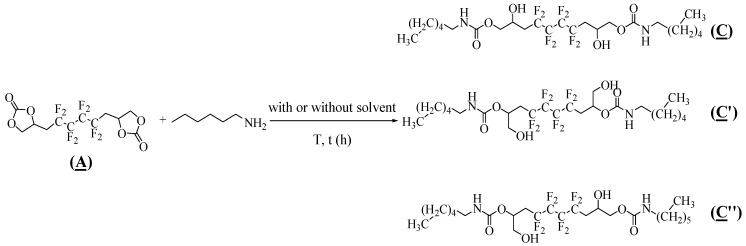
Synthesis of three possible regioisomer hydroxyurethanes by addition of hexylamine to (**A**).

The hydroxyurethanes synthesis conditions were investigated and optimized by adding hexylamine to the synthesized bis-CC (**A**). The model reaction was studied by varying the temperature, solvent, time, (**A**)/amine molar ratio, and the presence of a catalyst (Table 2). Due to the asymmetry present in (**A**), the nucleophilic attack of hexylamine on both carbonyl groups led to three possible regioisomers, including the hydroxyurethane groups: (i) symmetric isomer with secondary hydroxyl groups (**C**), (ii) symmetric isomer with primary hydroxyl groups (**C′**), and (iii) asymmetric isomer having both primary and secondary hydroxyl groups (**C**″) (Figure 4).

**Table 2:** Model reaction of (**A**) with hexylamine ^a^: conditions optimization.

Entry	Solvent	(A): Amine	T (°C)	t (h)	Cat. (2 mol%)	Conv. (%) ^b^	Yield (%) ^c^	Secondary Alcohol (%) ^d^	Primary Alcohol (%) ^d^
1	DMSO	1:2.0	25	48	-	55	48 ^e^	66	34
2	DMSO	1:2.0	60	18	-	72	57 ^e^	71	29
3	DMSO	1:2.0	80	18	-	81	55 ^e^	70	30
4	DMF	1:2.0	80	18	-	92	49 ^e^	73	27
5	Acetonitrile	1:2.0	80	18	-	79	52	65	35
6	Dimethyl carbonate	1:2.0	80	18	-	73	52	67	33
7	1,4-dioxane	1:2.0	80	18	-	85	48	70	30
8	Ethyl acetate	1:2.0	80	18	-	88	42	65	35
9	Trifluoro-toluene	1:2.0	80	18	-	56	50	68	32
10	-	1:2.0	80	18	-	59	48	65	35
11	-	1:2.6	80	18	-	70	64	60	40
12	-	1:3.0	80	18	-	100	69	65	35
13	-	1:2.0	80	5	NEt_3_	100	63	65	35
14	-	1:2.6	80	5	NEt_3_	100	84	68	32
15	-	1:3.0	80	5	NEt_3_	100	92	70	30

^a^ Conditions: carried out in a 10 mL flask; (**A**)/solvent (if any) 0.5 g/2 mL. ^b^ Determined by ^1^H NMR: Conv. % =(∫7.2NH (7,7′)2(∫7.2NH (7,7′)/2+∫4.6OCH2 (1,1′ of carbonate)/2 × 100; where $\int_{i}^{}XY$ stands for the integral of the signal centered at i ppm assigned to XY group (Figure 5 and Figure S1 in the Supplementary Materials). ^c^ The yield was calculated after purification. ^d^ The proportions of primary alcohols and secondary alcohols were calculated using ^1^H NMR spectra of the pure products: % secondary OH =(∫4.06CH (10,10′)1(∫4.06CH (10,10′)/1+∫5.07CH (13, 13′)/1 × 100; % primary OH =100−% secondary OH (Figure 5). ^e^ NMR reveals the presence of side products.

**Figure 5:**
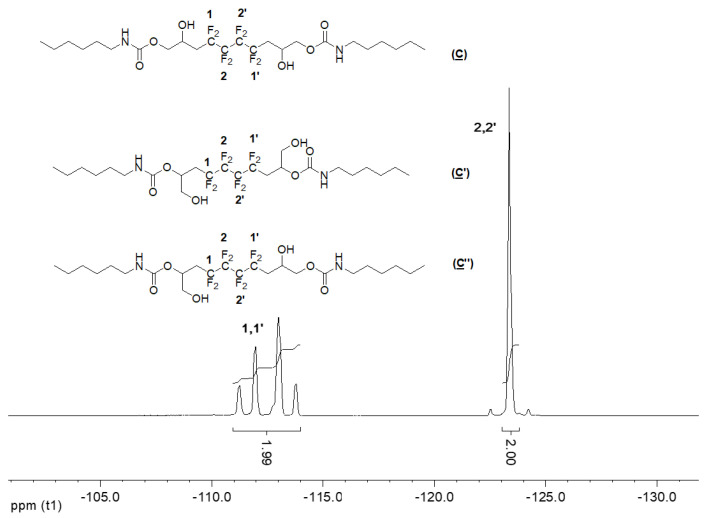
^1^H NMR spectrum of the model hydroxyurethanes (**C**), (**C′**), and (**C″**) (DMSO-d6, 20 °C, 400 MHz).

First, the reaction was attempted in the absence of a catalyst. 55% conversion was obtained upon reacting (**A**) with 2.0 eq hexylamine at 25 °C for 48 h (Table 2, Entry 1). Subsequently, the effect of the temperature was evaluated using 2.0 eq amine in DMSO. At 60 °C, 18 h of reaction time was enough to increase the conversion of (**A**) to 72% (Table 2, Entry 2), which kept rising to 81% at 80 °C (Table 2, Entry 3). Therefore, the evolution of this reaction is highly dependent on temperature, proving the need for thermal activation of the aminolysis of (**A**). Nevertheless, in the presence of DMSO, it was observed that these reactions led to side products, evidenced by the ^1^H NMR spectrum and confirmed by LC-MS chromatography. Similarly, this was seen when DMF was implemented in the same conditions (Table 2, Entry 4). The addition reaction was then attempted in different solvents (Table 1, Entry 5–9), leading to the model oligomers in unsatisfactory yields and low to moderate conversions with no side products detected. Then, we focused on investigating the reaction in bulk, where there was no sign of any side products detected by ^1^H NMR. In the absence of any catalyst, the viscosity of the medium increased with the progress of the reaction, and a conversion reached 70% after 18 h at 80 °C with 2.6 eq of hexylamine (Table 2, Entry 11). This viscosity can be linked to the formation of a new hydroxyl group, driving the creation of hydrogen bonds and decreasing the mobility of the reagents. Moreover, it was necessary to use 3.0 eq hexylamine to completely convert the cyclocarbonate (**A**) in 18 h reaction time (Table 1, Entry 12). This may be due to the better stirring obtained upon increasing the liquid phase. Finally, the reaction time was optimized by adding 2% triethylamine as a catalyst. A 100% conversion and 92% yield was reached after a 5 h reaction at 80 °C with 3.0 eq of hexylamine (Table 1, Entry 15). In all the studied entries, it was noted that there was no significant difference in the proportions of the hydroxyl groups; 65–73% of secondary alcohols were quantified, consistent with the reported literature [10,35].

### 2.3. Characterization of the Model Hydroxyurethanes

The model hydroxyurethanes (**C**), (**C′**), and (**C**″) were characterized by ^1^H, ^19^F, and ^13^C NMR spectroscopy. By comparing Figure 5 with the ^1^H NMR spectrum of the fluorinated bis-CC (**A**) (Figure S1 in the Supplementary Materials), it is observed that the AB system corresponding to the two nonequivalent hydrogen atoms CH_a_H_b_ of the carbonate groups has disappeared (at 4.6 and 4.2 ppm). Furthermore, the proton of the amide group (7, 7′) appeared at 7.2 ppm. The two peaks at 3.5 ppm (14, 14′) and 4.1 ppm (10, 10′) evidence the presence of the α-CH_2_ group of the primary and secondary alcohols, respectively. The multiplet appearing at 3.9 ppm (9, 9′) can be assigned to the carboxylate group’s α-CH_2_. The protons (12, 12′) in the CH_2_CF_2_ groups appeared as one multiplet (complex coupling with fluorine in positions α and β) at 2.2 ppm. In addition, the protons of the methyl (1, 1′) and methylene (2–5 and 2′–5′) groups, originally from the hexylamine, appear at 0.8 ppm and a multiplet at 1.3 ppm, respectively. The chemical shift at about 3 ppm can be attributed to the other two protons of the amine chain (6, 6′).

**Figure 6:**
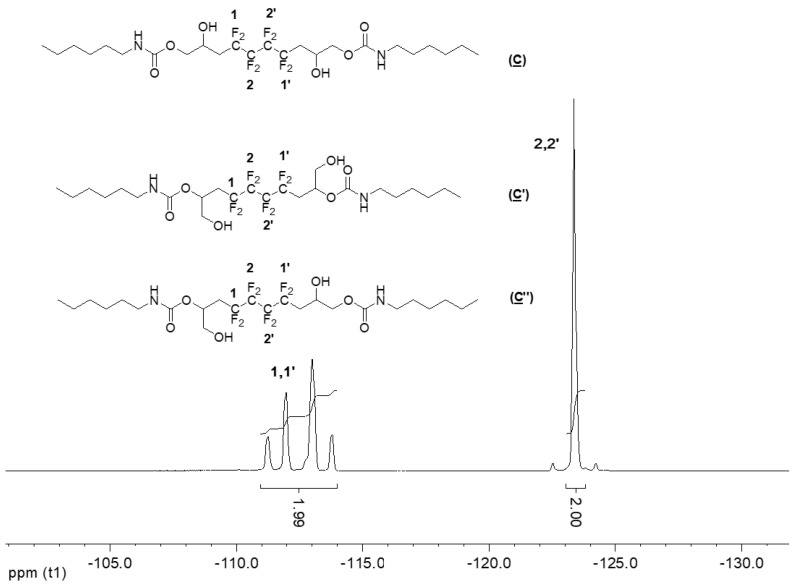
^19^F NMR spectrum of the model hydroxyurethanes (**C**), (**C′**), and (**C″**) (DMSO-d6, 20 °C, 235.2 MHz).

The ^19^F NMR spectrum of the hydroxyurethanes (Figure 6) is similar to that of (**A**) (Figure S2 in the Supplementary Materials). It shows the presence of an AB system located at δ − 112 ppm assigned to CH_2_CF_A_F_B_CF_2_ groups of the diastereoisomers due to the presence of the asymmetric carbon (10, 10′ and 13, 13′). Furthermore, the signal at δ − 123 ppm is assigned to the difluoromethylene groups (CH_2_CF_2_CF_2_).

**Figure 7:**
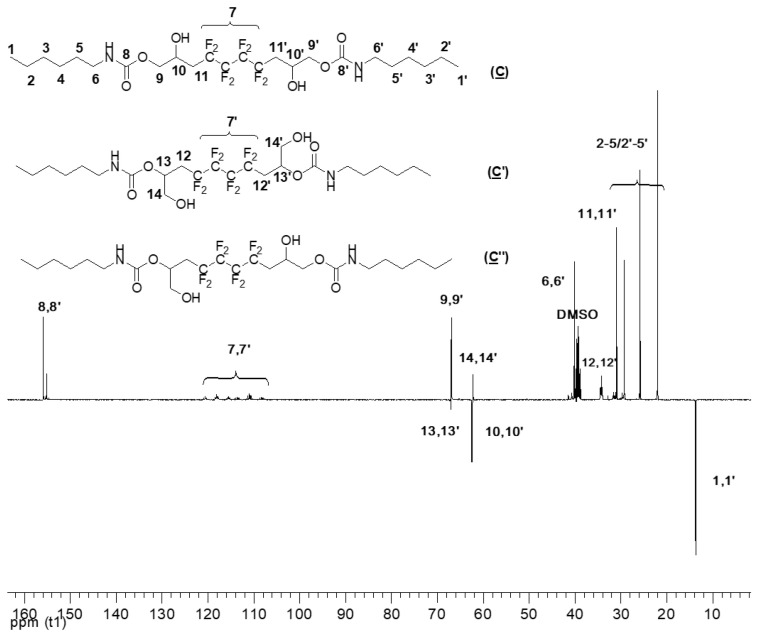
^13^C NMR spectrum of the model hydroxyurethanes (**C**), (**C′**), and (**C″**) (DMSO, 20 °C, 100.6 MHz).

Expectedly, the ^13^C NMR spectra (DEPT135) of (**C**), (**C′**), and (**C**″) (Figure 7) has many similar characteristics observed in (**A**) (Figure S3 in the Supplementary Materials). The signal at δ 155 ppm corresponds to the carbonyl groups (8, 8′). In addition, the signal of CF_2_ groups (7, 7′) appeared between δ 106 and δ 120 ppm. The carboxylate group’s α-CH_2_ (9, 9′) can be found at δ 67 ppm. The chemical shift at 31 ppm corresponds to the methylene group adjacent to CF_2_ groups (11, 11′). Most importantly, the signals of the carbon atoms bearing the hydroxyl group are characterized and distinguished: 10, 10′ hold the secondary OH, whereas 13, 13′ are the β-CH to the primary OH; they correspond to signals centered at 63 and 67 ppm, respectively. The low intensity of 13,13′ confirms the low amount of primary alcohol. As for the hexylamine used in the addition reaction, its presence as a part of the model products is highlighted by the peaks at 40 ppm, 22 ppm, and 30 ppm assigned to the CH_2_ adjacent to the amide (6, 6′) and CH_2_ groups of the amine (2–5/2′–5′), respectively. Finally, the signal of the CH_3_ (1, 1′) is noted at δ 14 ppm.

### 2.4. Synthesis and Characterization of the FPHUs and PHUs: Reaction with Diamine

**Figure 8:**
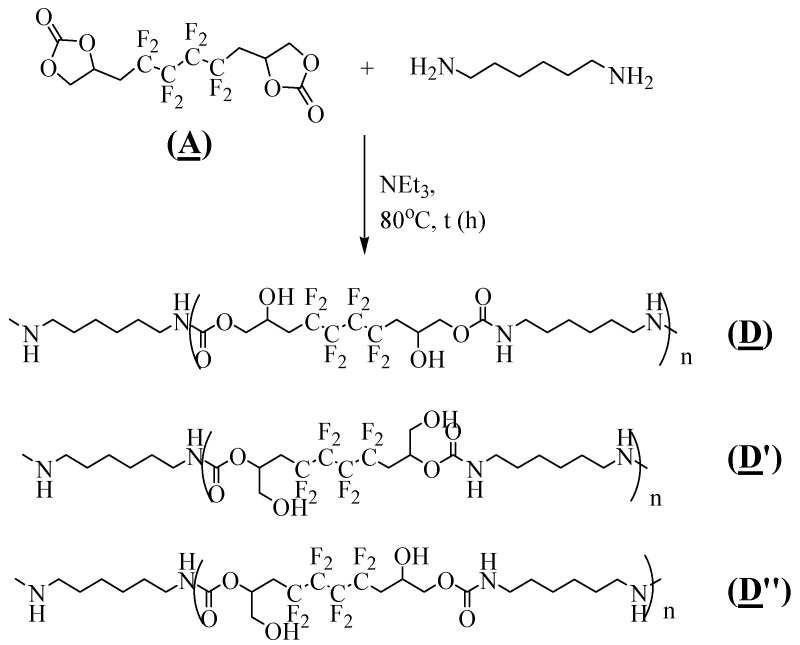
Synthesis of FPHUs by polyaddition of (**A**) with hexamethylenediamine.

The fluorinated polyhydroxyurethanes (FPHUs) were synthesized by ring opening of the fluorinated bis-CC (**A**) with different molar ratios of (**A**)/hexamethylenediamine, based on the optimized conditions obtained in the model reaction and taking into consideration the two active hydrogen atoms in the diamine groups (Figure 8).

A series of bulk polymerization reactions were conducted at 80 °C in the presence of 2% triethylamine, implementing 1.5, 1.3, and 1.0 eq of the diamine (Table 3). Initially, (**A**) was fully converted after adding 1.5 eq of the hexamethylenediamine to give FPHUs **P1** oligomers in 82% yield (Table 3, Entry 1). Then, a varying ratio of hexamethylenediamine was used in combination with (**A**) to produce FPHUs with different molar masses (Table 3, Entry 2–3). Adding 1.0 eq of hexamethylenediamine yielded FPHUs **P3** polymers in 61% yield and 94% conversion after 18 h reaction (Table 3, Entry 3). Finally, the synthesis of PHUs **P4** was performed to compare their properties to that of FPHUs. The hydrogenated polymers were synthesized using the same optimized conditions used in **P3** synthesis (Table 3, Entry 4). (**B**) was 88% converted by reacting it with 1.0 eq of hexamethylenediamine for 18 h. **P4** was produced in 57% yield, characterized, and confirmed via ^1^H NMR spectroscopy (Figure S6 in the Supplementary Materials). It is worth noting that the reactivity of the fluorinated bis-CC (**A**) was higher than the hydrogenated bis-CC (**B**) with the diamine under the same conditions.

**Figure 9:**
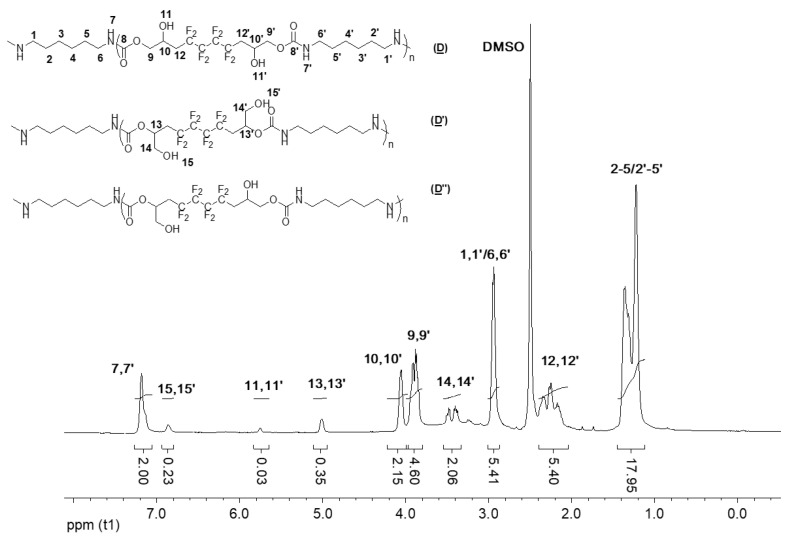
^1^H NMR spectrum of the synthesized **P3** (**D**), (**D′**), and (**D″**) (DMSO-d6, 20 °C, 400 MHz).

Next, the target FPHUs **P3** were characterized by NMR spectroscopy. The ^1^H NMR spectrum of the pure **P3** (Figure 9) is very similar to that of the products of the model reaction. The main difference is the absence of the CH_3_ signal of the terminal amine at 0.8 ppm.

**Figure 10:**
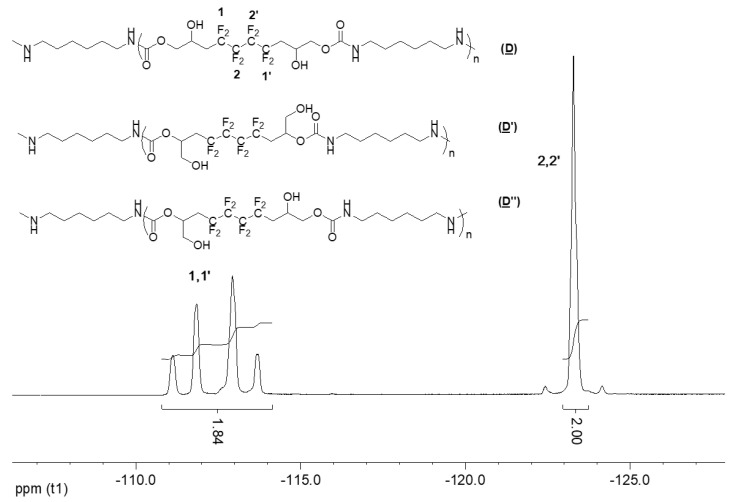
^19^F NMR spectrum of the synthesized **P3** (**D**), (**D′**), and (**D″**) (DMSO-d6, 20 °C, 235.2 MHz).

Comparably, the ^19^F NMR spectrum of **P3** (Figure 10) is similar to that of the products of the model reaction and the synthesized bis-CC (**A**) (Figure S2 in the Supplementary Materials), showing the presence of the AB system at δ − 112 ppm, and the signal at δ − 123 ppm.

**Figure 11:**
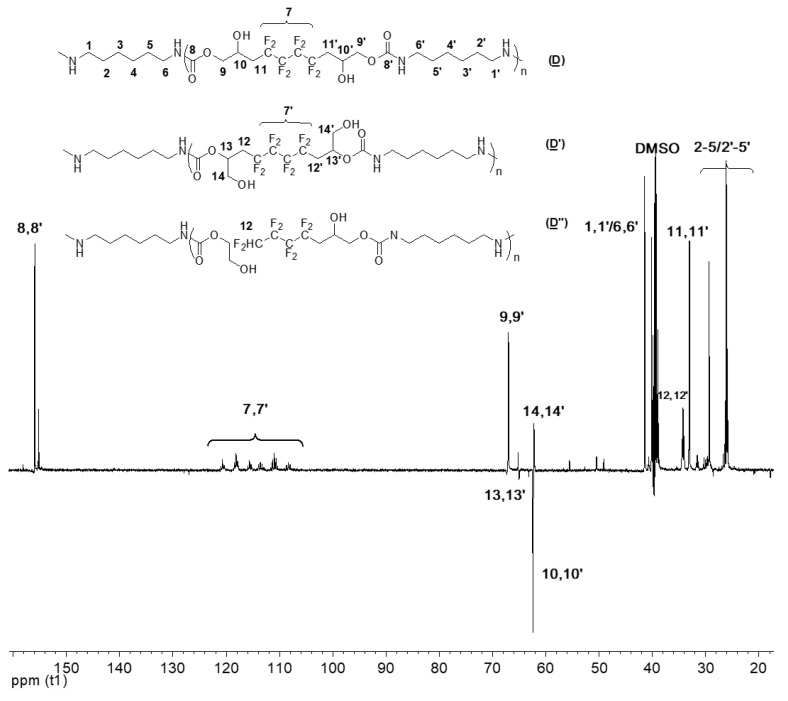
^13^C NMR (DEPT 135) spectrum of the synthesized **P3** (**D**), (**D′**), and (**D″**) (DMSO, 20 °C, 100.6 MHz).

In turn, the ^13^C NMR spectrum (DEPT135) of **P3** (Figure 11) revealed almost the same characteristics seen in the products of the model reaction, except for the absence of the CH_3_ peak of the amine.

### 2.5. Gel Permeation Chromatography and Thermal Analysis of FPHUs and PHUs: Comparison

The synthesized FPHUs and PHUs were characterized and compared by GPC, TGA, and DSC (Table 4).

This polymerization seemed highly dependent on the excess of the diamine initially implemented which is in accordance with the reported literature where the excess of diamine adjusted the degree of polymerization, and thus, their molar masses, based on Flory’s theory and Carothers’ equation [30,44]. For example, due to the stoichiometry used, **P1**, and **P2** could only reach maximum degrees of polymerization of 5 and 8 respectively (at 100% extent of reaction). The different CC/diamine molar ratios used in the polyaddition reflect the variation of the molar masses of FHPUs **P1**–**P3**; indeed, the degree of polymerization upon the polyaddition decreased as soon as the diamine was in excess (Table 4, Entry 1–2). Higher molar mass was obtained by implementing a stoichiometric CC/diamine ratio (Table 4, Entry 3). As reported by Besse et al. in 2015, the relatively low molar masses of the FPHUs obtained can be attributed to numerous side reactions at the early stages of the polyaddition reaction, yielding undesired compounds. By comparing the fluorinated and non-fluorinated polymers, **P3** and **P4**, FPHUs exhibited higher molar mass with 1.0 equivalent of the diamine, whereas the polydispersity indexes of both polymers were close (Table 4, Entry 3–4).

The thermogravimetric data gives an insight into the stability of the polymers and estimates the degradation’s initiation and completion required temperatures. The most relevant TGA results are listed in Table 4. Indeed, the degradation in **P3** started (T_5%_) at a higher temperature than in **P1** and **P2**. The complete degradation, estimated arbitrarily at 70 wt% loss, was observed at 450 °C in **P3**. By comparing the thermogravimetric results of both polymers FPHUs **P3** and PHUs **P4** (Figure S7), all the decomposition temperatures for **P4** were lower than in the fluorinated counter, typically by 50–80 °C (Table 4). Relatively low values of the glass transition temperature, T_g_, were observed for FHPUs **P1**–**P3** and PHUs **P4**. However, the latter shows a lower T_g_ than FPHUs. This can be explained by the larger atomic size of fluorine compared to hydrogen.

## 3. Materials and Methods

### 3.1. Materials

1,4-bis(2′,3′-epoxypropyl)perfluorobutane (BEPFB) was provided by TOSOH Finechemical Corporation (Shunan, Japan). Lithium bromide (LiBr), acetone (analytical grade), hexylamine, hexamethylenediamine, 1,2,7,8-diepoxyoctane, Dimethylformamide (DMF), Dimethyl Sulfoxide (DMSO), acetonitrile, ethyl acetate, dimethyl carbonate, trifluorotoluene, and 1,4-dioxane were purchased from Sigma-Aldrich. The deuterated solvents were purchased from Euroiso-top (purity > 99.8%).

### 3.2. Characterization

#### 3.2.1. Nuclear Magnetic Resonance (NMR)

The NMR spectra were recorded on a Bruker AC 400 instrument, using deuterated chloroform, d_6_-N,N-dimethylsulfoxide, and d_6_-acetone as solvents, and tetramethylsilane (TMS) (or CFCl_3_) as references for ^1^H (or ^19^F) nuclei. Coupling constants and chemical shifts are given in hertz (Hz) and parts per million (ppm), respectively. The experimental conditions for recording ^1^H, ^13^C, (or ^19^F) NMR spectra were as follows: flip angle 90° (or 30°), acquisition time 4.5 s (or 0.7 s), pulse delay 2 s (or 2 s), number of scans 128 (or 512), and a pulse width of 5 μs for ^19^F NMR.

#### 3.2.2. Thermogravimetric Analysis (TGA)

TGA was performed with a TGA 51 apparatus from TA Instruments, under air, and at the heating rate of 10 °C/min from room temperature up to a maximum of 550 °C. The sample weight varied between 10 and 15 mg.

#### 3.2.3. Differential Scanning Calorimetry (DSC)

DSC analysis was conducted on a Netzsch 200F3 DSC apparatus equipped with Proteus software under a nitrogen atmosphere at a heating rate of 20 °C/min. The temperature range was between −50 to +200 °C. The DSC system was first calibrated in temperature using indium and n-hexane. The second run led to the glass transition temperature, defined as the inflection point in the heat capacity jump. The sample weight was about 10 mg.

### 3.3. Synthetic Procedures

#### 3.3.1. Synthesis of Fluorinated Biscyclocarbonate (**A**)

##### 4-(2,2,3,3,4,4,5,5-octafluoro-6-(2-oxo-1,3-dioxolan-4-yl)hexyl)-1,3-dioxolan-2-one

The reactions were performed in an autoclave of 100 mL Parr Hastelloy. First, LiBr (276 mg, 3.18 mmol) dissolved in acetone (30 mL) was placed into the autoclave which was pressurized to 30 bars of nitrogen for 1 h to check for leaks. Once the nitrogen was evacuated, the reactor was placed under vacuum for 30 min, and then acetone (30 mL), 1,4-bis(2′,3′-epoxypropyl)perfluorobutane (10.02 g, 0.0318 mol), and carbon dioxide (15–20 bars) were added. The reactor was then heated progressively to 65 °C for 96 h, and the pressure and temperature evolution were recorded. Subsequently, the reactor was cooled and degassed (release of unreacted CO_2_), and the crude product was washed with acetone (100 mL), filtered, then dried under a vacuum.

^1^H NMR (400.1 MHz, DMSO, δ): 2.90 (m, CH_2_CF_2_, 4H); 4.63 and 4.22 (m, OCH_2_, 4H); 5.20 (m, CH, 1H).

^19^F NMR (235.2 MHz, DMSO, δ): −112.29 (AB system, CH_2_CF_A_F_B_CF_2_), −123.18 (s, CH_2_CF_2_C**F**_2_).

^13^C NMR (100.6 MHz, DMSO, δ): 154.38 (C=O); 106.56–120.72 (CF_2_); 70.24 (CH); 60.24 (OCH_2_); 39.26(**C**H_2_CF_2_).

#### 3.3.2. Synthesis of Non-Fluorinated Biscyclocarbonate (**B**): 4-(6-(2-oxo-1,3-dioxolan-4-yl)hexyl)-1,3-dioxolan-2-one

The carbonylation of the hydrogenated epoxide 1,2,7,8-diepoxyoctane was performed using the same procedure as the fluorinated epoxide using the following quantities and conditions: LiBr (605.3 mg, 6.97 mmol), DMF (30 mL), 1,2,7,8-diepoxyoctane (9.911 g, 0.0697 mol), and carbon dioxide (15–20 bars). The reactor was heated progressively to 65 °C for 96 h. The crude product was distilled under a high vacuum pump at 40–60 °C and then placed under direct vacuum overnight to remove the DMF. However, the DMF was not totally removed until the product was extracted in H_2_O/ethyl acetate.

^1^H NMR (400.1 MHz, DMSO, δ): 1.38 (m, CH_2_CH_2_, 4H); 1.69 (m, CH_2_CH, 4H); 4.56 and 4.12 (m, OCH_2_, 4H); 4.76 (m, CH, 1H).

^13^C NMR (100.6 MHz, DMSO, δ): 76.87 (CH); 69.15 (OCH_2_); 32.63(CHCH_2_CH_2_); 23.56(CH_2_CH_2_CH_2_).

#### 3.3.3. Model Reaction of the Fluorinated Bis-CC (**A**) with Monoamine: Optimization of the Reaction Conditions

(**A**) (2.00 g, 4.97 mmol, 1.00 eq), hexylamine (2.00, 2.60, or 3.00 eq), and triethylamine (NEt_3_) (10 mg, 2–3 drops) were placed in a round bottom flask. The flask was heated to 80 °C to dissolve (**A**). The mixture was then stirred, and a sample was taken at t_0_. The flask was then placed in an oil bath at the desired temperature for a specific time (t_h_). Finally, the model product (**C**, **C′**, and **C**″) was washed with distilled water and pentane, water was removed by decantation, and the material was dried at 60 °C under vacuum for 16 h.

^1^H NMR (400.1 MHz, DMSO, δ): 0.90 (m, CH_3_CH_2_, 6H); 1.18 and 1.35 (m, CH_2_, 16H); 2.22 (m, CH_2_, 4H); 2.9 (m, CH_2_NH, 4H); 3.45 (m, CH_2_OH, 4H); 3.9 (m, CHCH_2_O, 4H); 4.05 (s, CHOH, 2H); 5 (s, OCHCH_2_, 2H); 5.3 (s, CHOH, 2H); 6.8 (s, CH_2_OH, 2H); 7.15 (m, NHCOO, 2H).

^19^F NMR (235.2 MHz, DMSO, δ): −112.39 (AB system, CH_2_CF_A_F_B_ CF_2_); −123.36 (s, CH_2_CF_2_CF_2_).

^13^C NMR (100.6 MHz, DMSO, δ): 155.59 (C=O); 107.75–120.95 (CF_2_); 66.96 (OCH_2_); 62.26 (CH_2_OH); 63.04/63.27 (CHOH/ CH_2_O); 40.16 (CH_2_NH); 30.91 (**C**H_2_CF_2_); 21.99–29.28 (CH_2_CH_2_); 14.46 (**C**H_3_CH_2_).

#### 3.3.4. The Reaction of the Fluorinated Bis-CC (**A**) with a Diamine: Formation of the FPHUs (**D**, **D′**, and **D″**)

Similarly to the model reaction, using the following quantities: (**A**) (1.00 g, 2.48 mmol, 1.00 eq), hexamethylenediamine (1.00, 1.30, or 1.50 eq), and triethylamine (NEt_3_) (5 mg).

^1^H (400.1 MHz, DMSO, δ): 1.21–1.37 (m, CH_2_CH_2_); 2.24 (m, CH_2_CF_2_); 2.94 (m, CH_2_NH); 3.44 (m, CH_2_OH); 3.88 (m, CHCH_2_O); 4.05 (s, CHOH); 5 (s, OCHCH_2_); 5.74 (s, CHOH); 6.86 (s, CH_2_OH); 7.19 (m, NHCOO).

^19^F NMR (235.2 MHz, DMSO, δ): −112.5 (AB system, CH_2_CF_A_F_B_ CF*_2_*); −123.29 (s, CH_2_CF_2_CF_2_).

^13^C NMR (100.6 MHz, DMSO, δ): 155.94 (C=O); 106.74–121.03 (CF_2_), 67.00 (OCH_2_CH); 62.93/ 63.17 (CH_2_CHOH/OCHCH_2_); 62.21 (CH_2_OH); 41.43 (CH_2_NH); 33.04 (CH_2_CF_2_); 25.84–29.36 (CH_2_).

#### 3.3.5. The Reaction of the Non-Fluorinated Bis-CC (**B**) with a Diamine: Formation of the PHUs (**E**, **E′**, and **E″**)

Similarly to the synthesis of the above FPHUs, the following quantities were used: (**B**) (0.53 g, 2.33 mmol, 1.00 eq), hexamethylenediamine (0.270 g, 2.33 mmol, 1.0 eq), and triethylamine (NEt_3_) (10–12 mg).

^1^H NMR (400.1 MHz, DMSO, δ): 0.85 (m, CH_3_CH_2_, 6H); 1.22 to 1.53 (m, CH_2_, 24H); 2.93 (m, CH_2_NH, 4H); 3.37 (m, CH_2_OH, 4H); 3.54 (m, CHO, 2H); 3.78 (m, CHOH, 2H); 4.55 (m, CHCH_2_OH, 2H); 6.64 (s, CH_2_OH, 2H); 6.68 (s, CH_2_OH, 2H); 7.03 (m, NHCOO, 2H).

## 4. Conclusions

FPHUs were synthesized via the polyaddition of a synthesized fluorinated bis(cyclocarbonate) (bis-CC) with a telechelic diamine. First, the bis-CC was prepared by carbonylation of a fluorinated telechelic bisepoxide. Then, model additions of n-hexylamine onto the fluorinated CC were optimized; complete conversion and good yield were obtained when a 3-fold excess of hexylamine was used in bulk, catalyzed by NEt_3_. All attempts showed that secondary and primary alcohols were produced with about two-thirds of the former isomer. Polyaddition of the fluorinated bis-CC with hexamethylenediamine was conducted using the optimized conditions. FPHUs with various molar weights were formed using different CC/diamine molar ratios. The synthesized FPHUs were deeply characterized by NMR spectroscopy, and the PHUs were synthesized using the same optimized conditions and characterized for comparison purposes. The data collected by GPC, TGA, and DSC led to the same conclusion; the presence of the fluorinated chains provided a slightly higher thermal stability to the resulting polymers.

## Figures and Tables

**Table 1:** Syntheses of (**A**) and (**B**) by carbonylation of the corresponding diepoxides ^a^.

Entry	Diepoxide	Yield (%)
1	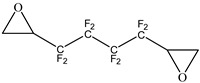	87
2	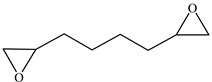	70

^a^ Reaction conditions: LiBr (5% mol), acetone, 96 h, 65 °C. P (CO_2_) = 15–20 bars, where CO_2_ was introduced to the autoclave repeatedly to maintain a constant pressure.

**Table 3:** FPHUs and PHUs synthesis from (**A**) or (**B**) with hexamethylenediamine ^a^.

Run#	CC	A: Diamine	Conv. (%)	Yield (%) ^b^	Secondary Alcohol (%) ^c^	Primary Alcohol (%) ^c^
FPHU **P1**	**A**	1:1.5	100	82	78	22
FPHU **P2**	**A**	1:1.3	100	66	71	29
FPHU **P3** ^d^	**A**	1:1.0	94	61	70	30
PHU **P4** ^d^	**B**	1:1.0	88	57	68	32

^a^ Conditions: Bulk polymerization, 2% of NEt_3_, 80 °C, 5 h. ^b^ The yield was calculated after purification, and the residual excess diamine was removed at the end of the reaction by distillation under a vacuum. ^c^ The proportions of primary and secondary alcohols were calculated using ^1^H NMR spectra of the pure products. ^d^ 18 h reaction time.

**Table 4:** Comparison of the properties of FPHUs and PHUs.

Entry	Polymer	M_n_ (g/mol) ^a^	PDI ^a^	T_g_ (°C) ^b^	T_5%_ (°C) ^c^	T_10%_ (°C) ^c^	T_70%_ (°C) ^c^
1	**P1**	3500	1.29	11.0	150	180	380
2	**P2**	5700	1.25	12.0	160	210	390
3	**P3**	9600	1.16	14.6	200	280	450
4	**P4**	6100	1.14	10.4	150	200	400

^a^ Number average molecular weight and polydispersity index were assessed by Gel Permeation Chromatography (GPC). ^b^ Determined by Differential Scanning Calorimetry (DSC). ^c^ Assessed by ThermoGravimetric Analysis (TGA), under air; 10 °C/min.

## Data Availability

The authors confirm that the data supporting the findings of this study are available within the article and its Supplementary Material.

## Supplementary Materials

The following supporting information can be downloaded at: https://www.mdpi.com/article/10.3390/molecules28041795/s1, Figure S1: ^1^H NMR spectra of (**A**) (top) and BEPFB (bottom) (DMSO-d6, 20 °C, 400 MHz); Figure S2: ^19^F NMR spectra of (**A**) (top) and BEPFB (bottom) (DMSO-d6, 20 °C, 235.2 MHz); Figure S3: ^13^C NMR (DEPT 135) spectra of (**A**) (top) and BEPFB (bottom) (DMSO, 20 °C, 100.6 MHz); Figure S4: ^1^H NMR spectrum of (**B**) (top) and 1,2,7,8-diepoxyoctane (bottom) (DMSO-d6, 20 °C, 400 MHz); Figure S5: ^13^C NMR (DEPT 135) spectrum of (**B**) (top) and 1,2,7,8-diepoxyoctane (bottom) (DMSO, 20 °C, 100.6 MHz); Figure S6: ^1^H NMR spectrum of the synthesized PHUs (DMSO-d6, 20 °C, 400 MHz); Figure S7: TGA thermograms of **P3** and **P4**.

## Sample Availability

Samples of the compounds are not available from the authors.
