# Supplementary material for: Original Fluorinated Non-Isocyanate Polyhydroxyurethanes
Source: Molecules. 2023 Feb 14;28(4):1795. doi: 10.3390/molecules28041795 (PMC9964802; doi:10.3390/molecules28041795)
Supplement: Supplementary file 1 [file molecules-28-01795-s001.zip › molecules-2208300-supplementary.pdf]

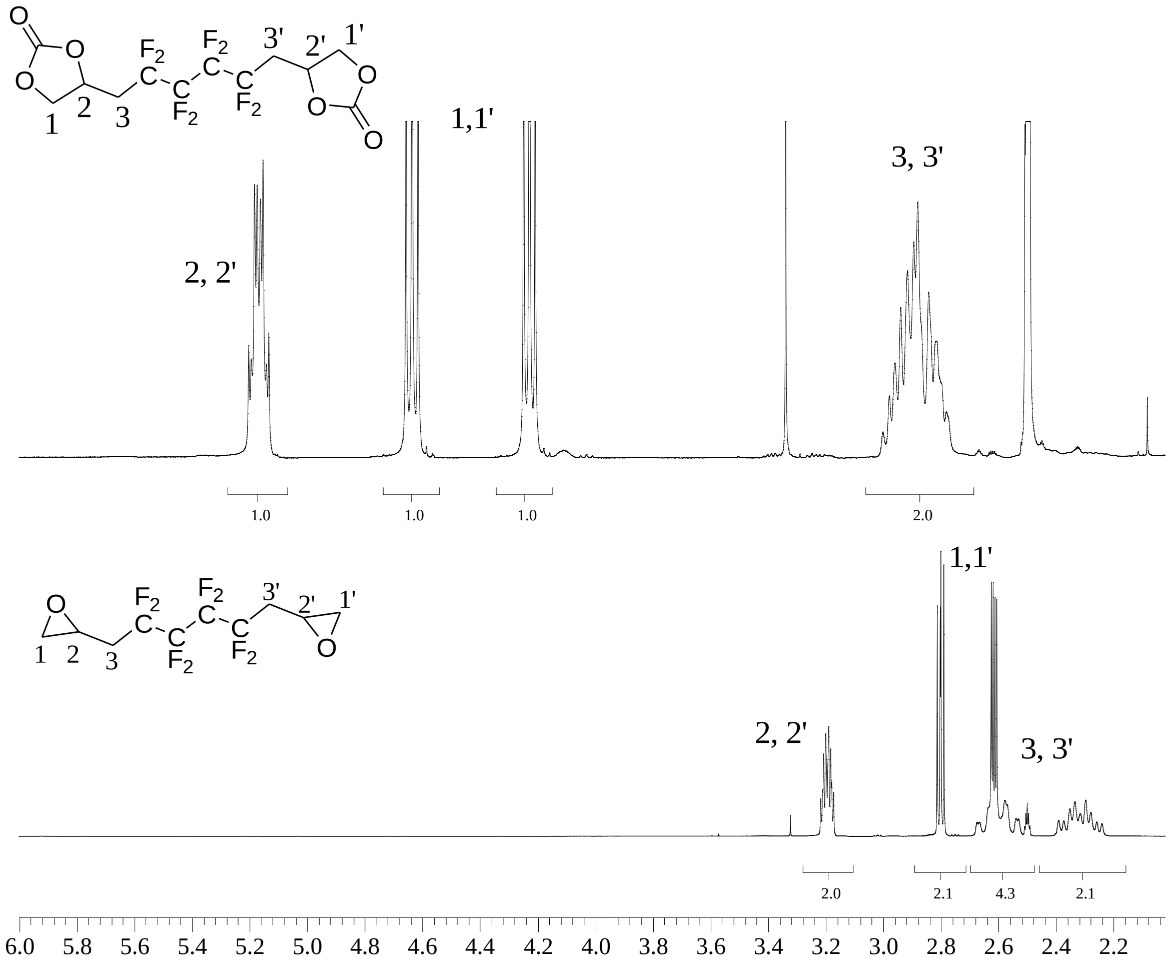


**Figure S1.** $^{1}$H NMR spectra of (**A**) (top) and BEPFB (bottom) (DMSO-d6, 20 °C, 400 MHz)

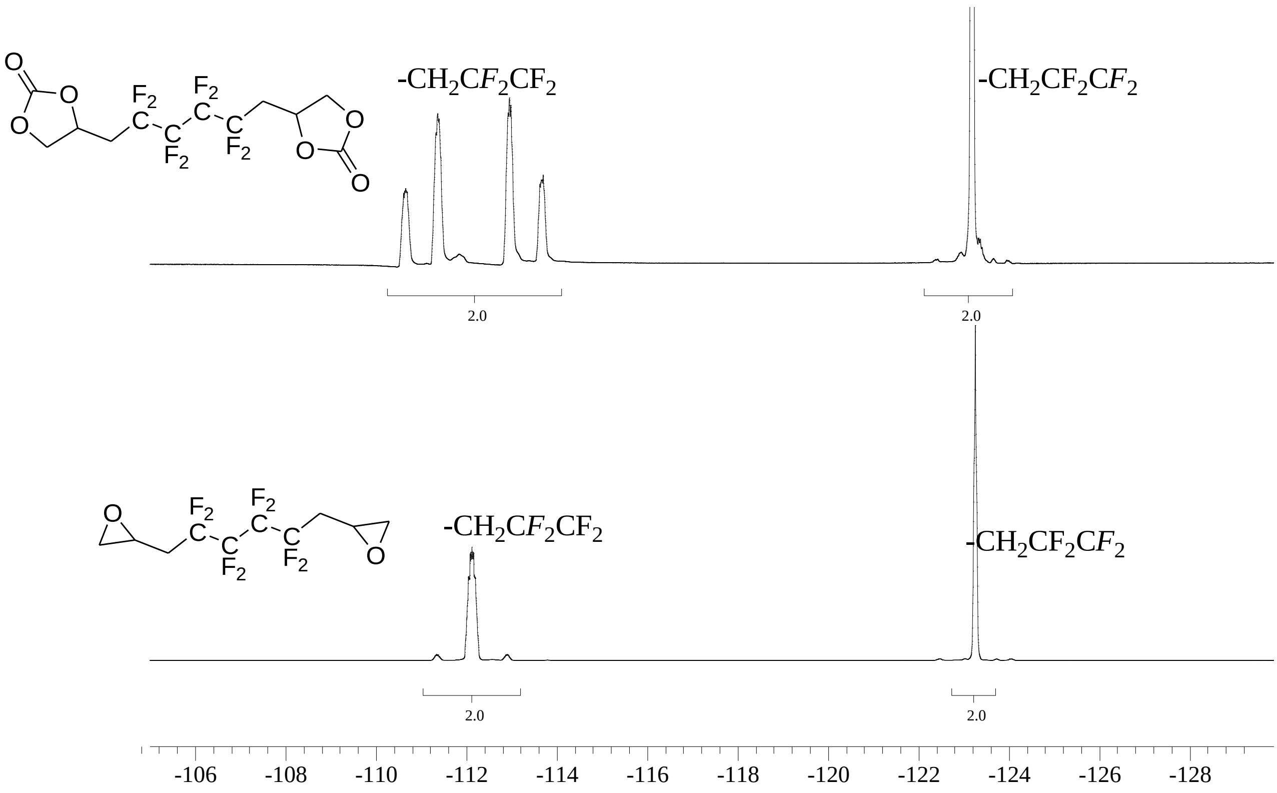


**Figure S2.** $^{19}$F NMR spectra of (**A**) (top) and BEPFB (bottom) (DMSO-d6, 20 °C, 235.2 MHz)

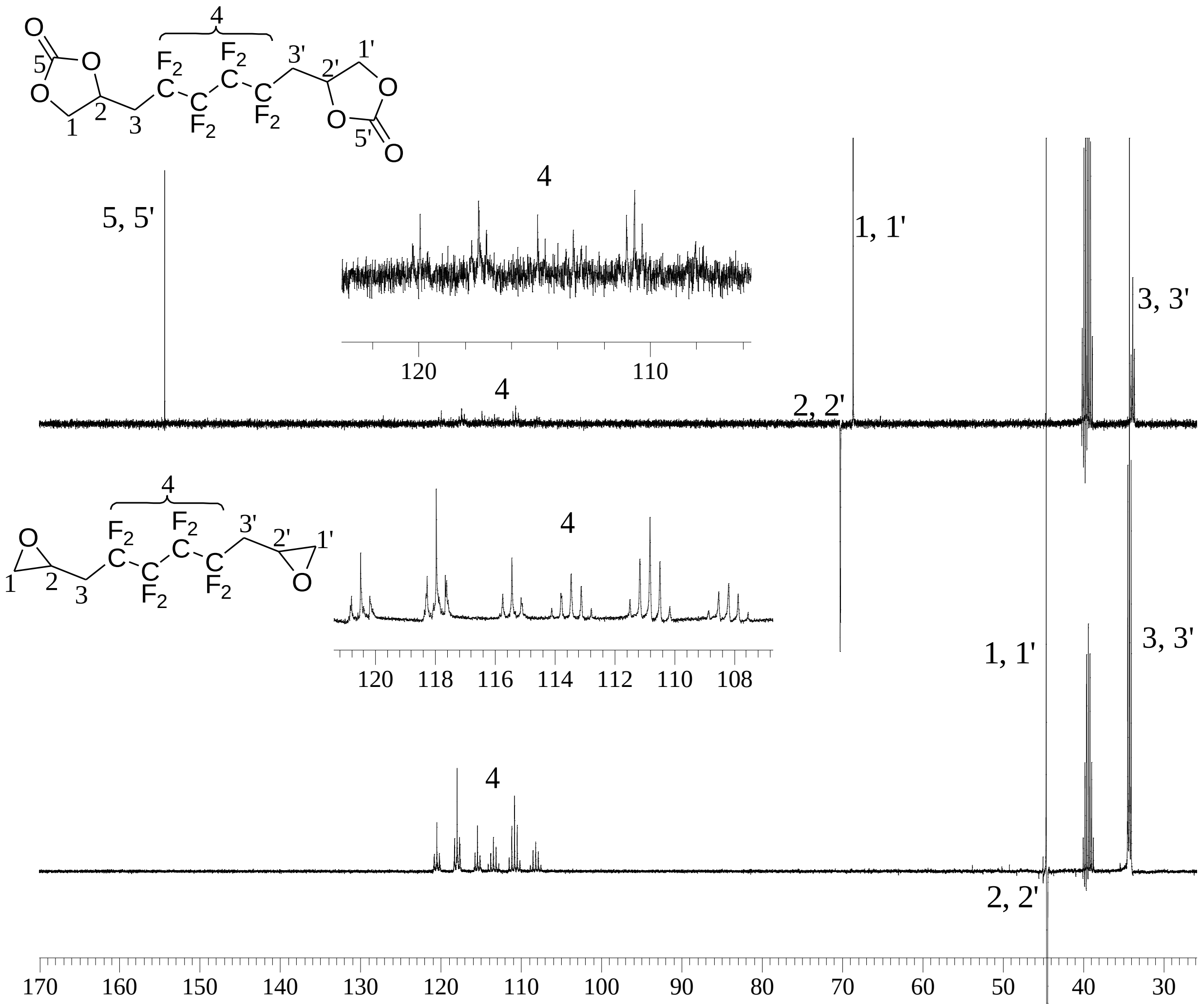


**Figure S3.** $^{13}$C NMR (DEPT 135) spectra of (**A**) (top) and BEPFB (bottom) (DMSO, 20 °C, 100.6 MHz)

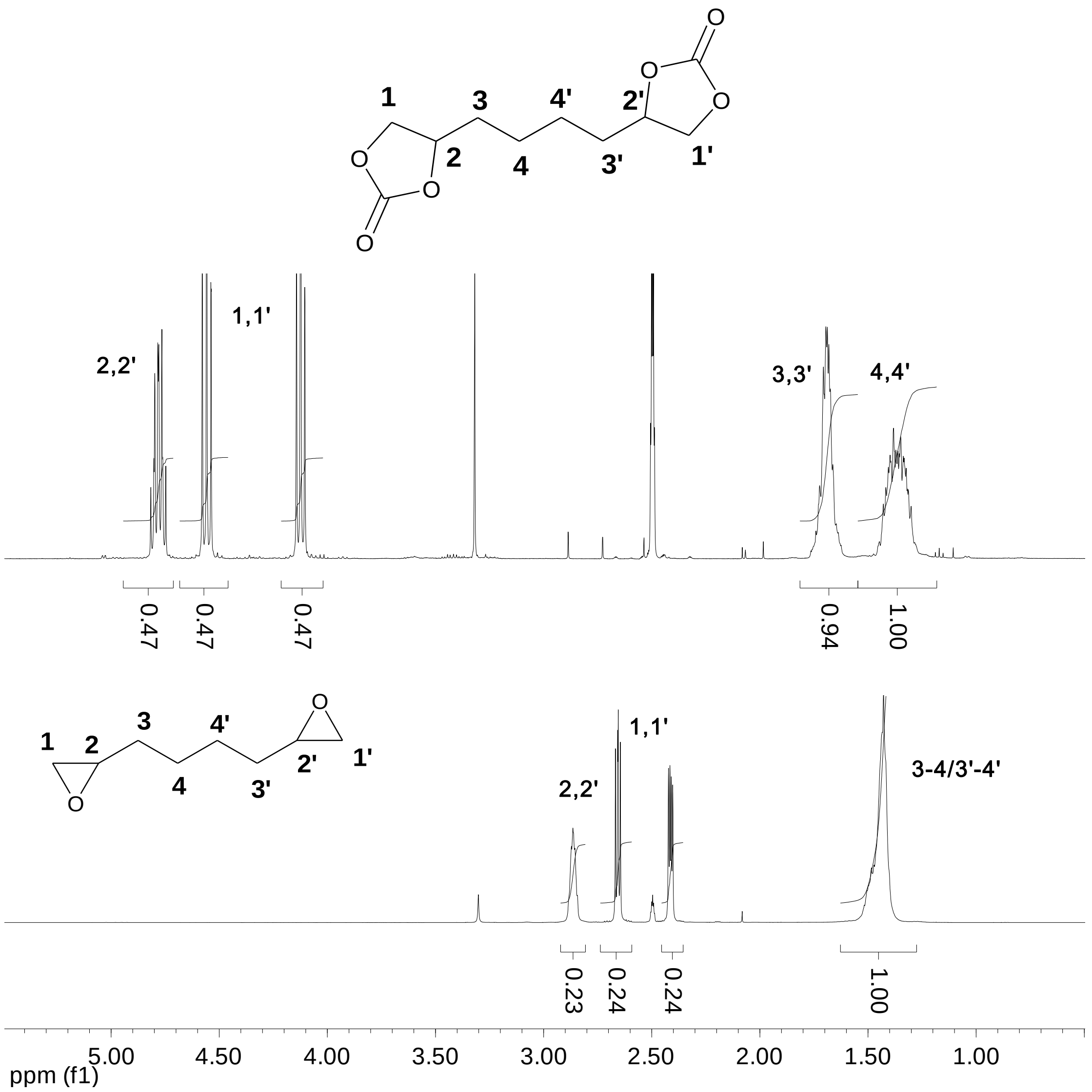


**Figure S4.** $^{1}H$ NMR spectrum of (**B**) (top) and 1,2,7,8-diepoxyoctane (bottom) (DMSO-d6, 20 °C, 400 MHz)

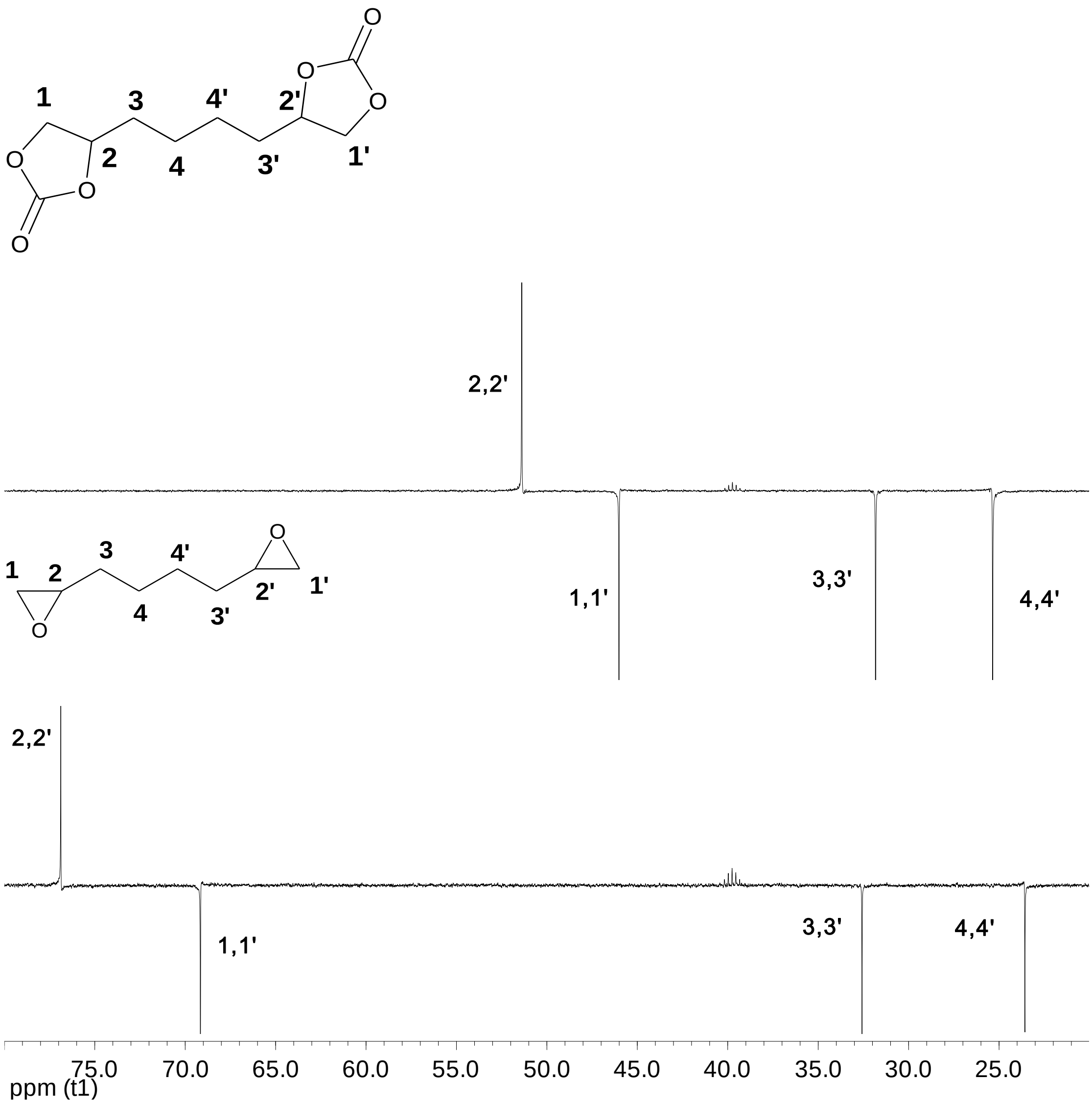


**Figure S5.** $^{13}$C NMR (DEPT 135) spectrum of (**B**) (top) and 1,2,7,8-diepoxyoctane (bottom) (DMSO, 20 °C, 100.6 MHz)

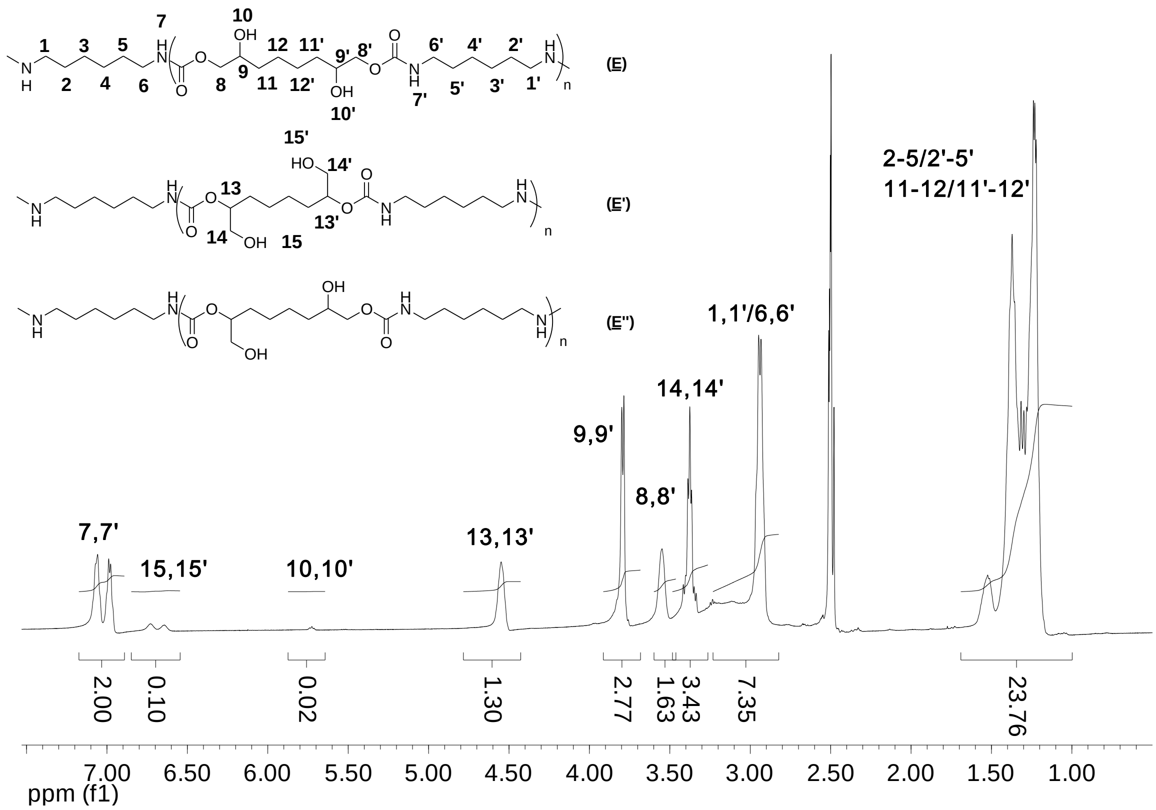


**Figure S6.** $^{1}H$ NMR spectrum of the synthesized PHUs (DMSO-d6, 20 °C, 400 MHz)

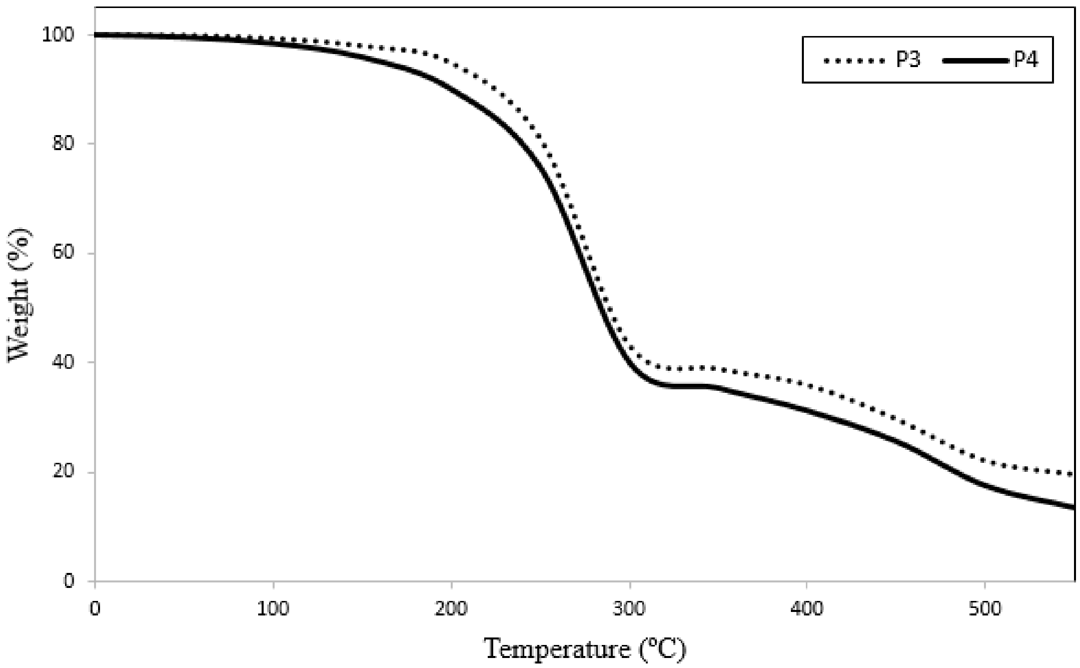


**Figure S7.** TGA thermograms of **P3** and **P4**
